# Association Between Serum Vitamin C and Non-alcoholic Fatty Liver Disease: A Cross-sectional Study

**DOI:** 10.5152/tjg.2022.21929

**Published:** 2023-02-01

**Authors:** Maodong Guo, Xiaohua Ye, Xin Shi

**Affiliations:** Department of Gastroenterology, Jinhua Hospital of Zhejiang University, Jinhua, Zhejiang, China

**Keywords:** NHANES, non-alcoholic fatty liver disease, vitamin C

## Abstract

**Background:**

The association between vitamin C and the risk of developing non-alcoholic fatty liver disease remains controversial. The aim of the present study is to examine any correlation between serum vitamin C and the risk of non-alcoholic fatty liver disease.

**Methods:**

Our study enrolled 3374 participants aged ≥ 20 years from the National Health and Nutritional Survey (2003-2006). Non-alcoholic fatty liver disease was defined as the US Fatty Liver Index ≥ 30 in the absence of other chronic liver disease. Multivariate logistic regression and the fitted smoothing curves were adopted for analyzing the correlation between serum vitamin C levels and the risk of developing non-alcoholic fatty liver disease.

**Results:**

After adjusting for all the covariates, it was discovered that serum vitamin C was negatively correlated with the risk of non-alcoholic fatty liver disease (odds ratio: 0.664, 95% CI: 0.512-0.860, *P* = .002). Through smooth curve fitting, it was further noticed that the relationship between serum vitamin C and the risk of non-alcoholic fatty liver disease was non-linear. The inflection point was 0.92, and to its left, a negative correlation was seen between vitamin C and non-alcoholic fatty liver disease (odds ratio: 0.451, 95% CI: 0.288-0.706, *P* = .001). To the right of the inflection point, however, the correlation between vitamin C and non-alcoholic fatty liver disease was not found to be significant.

**Conclusion:**

The correlation was non-linear between serum vitamin C levels and the risk of developing non-alcoholic fatty liver disease. Serum vitamin C was negatively correlated with the risk of non-alcoholic fatty liver disease when its level was less than 0.92 mg/dL.

**Main Points**

- Non-alcoholic fatty liver disease (NAFLD) is a major global health threat due to its growing incidence and prevalence. Moreover, NAFLD can progress to non-alcoholic steatohepatitis, cirrhosis, and primary hepatocellular cancer. Therefore, its prevention and treatment are of strong public interest.
- Evidence regarding the association between serum vitamin C levels and the risk of developing NAFLD was limited.
- We found that the correlation between serum vitamin C levels and the risk of developing NAFLD was non-linear. Serum vitamin C was negatively correlated with the risk of NAFLD when its level was less than 0.92 mg/dL.

## Introduction

Non-alcoholic fatty liver disease (NAFLD) is a manifestation of metabolic syndrome in the liver linked with obesity, type 2 diabetes mellitus, insulin resistance (IR), and dyslipidemia.^1^ Non-alcoholic fatty liver disease has quickly become one of the most common causes of liver disease globally affecting more than one-third of the general population. According to the latest meta-analysis, NAFLD was estimated to affect approximately 25% of the world population^2^ and is expected to rise to more than 100 million in 2030.^3^ Moreover, the prevalence of NAFLD in a Turkish region reached 60.1%, which was remarkable.^4^ This rapid rise is particularly concerning as NAFLD is a disease that often progresses and causes secondary complications including hepatocellular carcinoma and liver cirrhosis, and it is also a major factor behind liver disease-related morbidity.^5^ As a consequence of the serious complications and comorbidities, as well as the vast population affected, NAFLD is very expensive for the healthcare system, with estimated direct annual costs of over $100 billion in the United States.^6^ Given the magnitude of these challenges, there is an urgent need to identify interventions that can reduce NAFLD incidence and progression.

Vitamin C is a scavenger that is soluble in water, and it is capable of removing almost all free radicals relevant to the human body.^7^ Along with a few other species, humans cannot synthesize vitamin C, which must instead be acquired through diet. It has been shown in cross-sectional population-based studies that approximately 10%-20% of the Western population could have vitamin C deficiency, which is linked with many metabolic diseases and an elevated prevalence of all-cause mortality^8,9^ as NAFLD is commonly perceived as the hepatic representation of a metabolic syndrome. Given that vitamin C was found to be negatively correlated with metabolic syndrome,^10,11^ the potential effect of vitamin C on NAFLD has received increasing attention. In the past few years, a growing number of epidemiology research have been trying to explore the correlation between vitamin C and the risk of developing NAFLD, but no consensus has been established. Some literature have demonstrated that a low daily intake of vitamin C is linked with NAFLD, but other studies did not reach a conclusion.^12-16^ One plausible explanation for these contradicting results could be that most of those studies only evaluated the dietary intake of vitamin C instead of serum vitamin C concentrations. It has been established that dietary vitamin C intake has only a moderate correlation with serum vitamin C levels.^17^ Moreover, dietary intake of vitamin C does not reflect individual variations in the absorption, metabolism, tissue distribution, and other genetic disparities leading to different levels of circulating vitamin C. Therefore, serum evaluation might be more appropriate in determining the vitamin C levels in our subjects.

Hence, the primary aim of the present study is to describe the correlation between serum vitamin C concentrations and risk of developing NAFLD in a large cross-sectional population of adults in the United States, using the National Health and Nutrition Examination Survey (NHANES) data between 2003 and 2006.

## Materials and Methods

### Study Population

**Figure 1.**
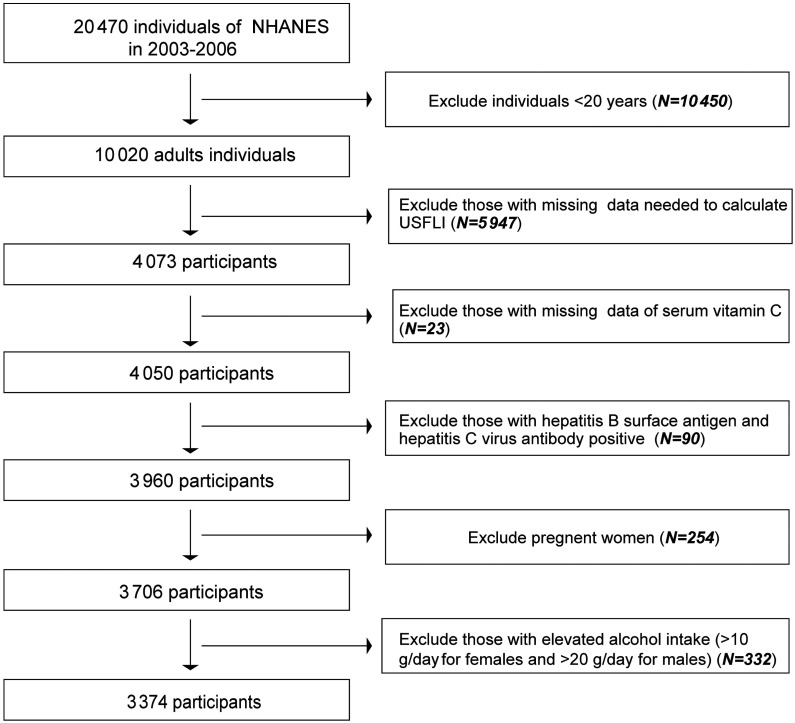
Flow chart of the study.

The data analyzed was obtained from the NHANES, which is a complex, stratified, multistage probability sample of the non-institutionalized US population. Four years of data across 2 NHANES cycles (2003-2004 and 2005-2006) were combined for analysis. The detailed information about the survey, laboratory, and examination procedures is accessible on the internet (www.cdc.gov/nchs/nhanes/). A total of 20 470 participants were identified initially; however, only participants aged 20 years and over were recruited for the present analysis. Among them, 17 096 participants were further excluded as they qualified one or more of the following criteria: (1) elevated alcohol intake (>10 g per day in females and >20 g per day in males); (2) lost information to determine the United States Fatty Liver Index (USFLI); (3) missing data of serum vitamin C; (4) participants who had positive hepatitis B surface antigen and/or hepatitis C virus antibodies; and (5) pregnant females. After excluding individuals who fall into the categories above, a total of 3374 participants were eligible for this study (Figure 1). The research ethical review board of the National Center for Health Statistics had approved all protocols, and all participants completed written informed consents.

### Measurement of Serum Vitamin C

In the 2003-2006 NHANES, serum vitamin C concentrations were tested at the Centers for Disease Control and Prevention in Atlanta. Before the test, all collected serum specimens were acidified, stabilized, and stored at −20°C. Serum vitamin C levels were quantified using the isocratic high-performance liquid chromatography (HPLC) with electrochemical detection.

### Definition of Non-alcoholic Fatty Liver Disease

The present study used the USFLI to define NAFLD. United States Fatty Liver Index is a non-invasive diagnostic index which has been extensively used for liver disease detection.^18-20^ This index incorporates age, race, fasting insulin, waist circumference, γ-glutamyltransferase, and fasting glucose. The equation of USFLI was defined as follows: USFLI = (e^− (e fas × non-Hispanic Black + 0.3458 × Mexican American + 0.0093 × age + 0.6151 × log^_e_^(GGT) + 0.0249 × waist circumference + 1.1792 × log^_e_^(insulin) + 0.8242 × log^_e_^(glucose) −glucose)^)/(1 + e^− e1cose× non-Hispanic Black + 0.3458 × Mexican American + 0.0093 × age + 0.6151 × log^_e_^(GGT) + 0.0249 × waist circumference + 1.1792 × log^_e_^(insulin) + 0.8242 × log^_e_^(glucose) −glucose)^) × 100. United States Fatty Liver Index score of ≥30 was assumed to have NAFLD.^19^

### Covariates

Covariates were selected based on known confounders from prior literature and clinical experience. Demographic variables of age, sex, race/ethnicity, the family poverty-to-income ratio (PIR), and education level were obtained from questionnaires. Family PIR was calculated as the annual household income divided by the federal poverty level and categorized into poverty (PIR ≤ 1) and beyond the poverty threshold (PIR > 1). Education levels were classified into below high school, high school level, and tertiary education above high school level. Smoking habit of patients was assessed as never smoked, current smoker, and former smoker. Patient characteristics including their weight, height, waist circumference, and blood pressure were recorded by trained interviewers using standardized procedures with calibrated equipment at the mobile examination center. Body mass index was calculated as weight (kg) divided by the square of the height (m^2^) and classified as normal weight (<25), overweight (25-<30), and obese (≥30) using the Centers for Disease Control and Prevention guideline. Laboratory covariates included total cholesterol, triglycerides, vitamin E (alpha-tocopherol), and vitamin A (retinol). Triglycerides and total cholesterol were measured enzymatically. Serum vitamin A and vitamin E levels were detected by HPLC with photodiode array detection. A glycated hemoglobin level ≥6.5%, a fasting blood glucose level ≥126 mg/dL, current use of anti-diabetic drugs, and/or self-reported diabetes diagnosis of patients were all defined as diabetes mellitus. Patient’s history of hypertension was established based on self-reported history of hypertension diagnosis, current use of prescription drugs for hypertension, a diastolic blood pressure ≥ 80 mmHg, and/or systolic blood pressure ≥ 140 mmHg.

### Statistical Analysis

Normally distributed continuous variables were expressed in the form of mean ± standard deviation and those with skewed distribution were represented by medians (interquartile ranges). Categorical variables were expressed as frequency or percentages. Independent *t*-test (normal distribution) or Mann–Whitney test (skewed distribution) and Pearson chi-squared test (categorical variables) were performed between individuals with or without NAFLD, as appropriate. Multiple logistic regression models were applied to inspect the association of serum vitamin C with NAFLD. Both unadjusted and adjusted models were performed, and odds ratios (ORs) with 95% CIs were calculated. In the adjusted regression model, the potential confounders include age, sex, race, PIR, smoking habit, education level, BMI, diabetes, hypertension, total cholesterol, triglycerides, vitamin A, and vitamin E. The non-linear or threshold effect of vitamin C on NAFLD was examined using a 2-piecewise linear regression with a smoothing function after adjusting for potential confounders. Stratified and interaction analyses were also conducted based on age groups (20-44, 44-65, ≥65 years), sex, race, family PIR, education level, smoking status, BMI, hypertension, and diabetes. All analyses were carried out using statistical package R (version 3.6.3; http://www.r-project.org) and EmpowerStats software (www.empowerstats.com, X&Y Solutions, Inc., Boston, Mass, USA). Statistical significance was set at a 2-tailed *P* of less than .05.

## Results

### Patient Characteristics

According to the exclusion and inclusion criteria, 3374 participants were enrolled eventually for further data analysis in our study. Table 1 demonstrates the comparison of basic characteristics of patients with and without NAFLD, which shows significant differences between groups, with the exception of the family PIR. The subjects with NAFLD tend to be older; were more likely to be male; be Mexican American; be former smokers; had hypertension and diabetes mellitus; had less than an education level above high school; had higher BMI, waist circumference, serum total cholesterol, triglycerides, vitamin A, vitamin E, gamma-glutamyltransferase, fasting insulin, and fasting glucose.

### Association of Serum Vitamin C with NAFLD

**Figure 2.**
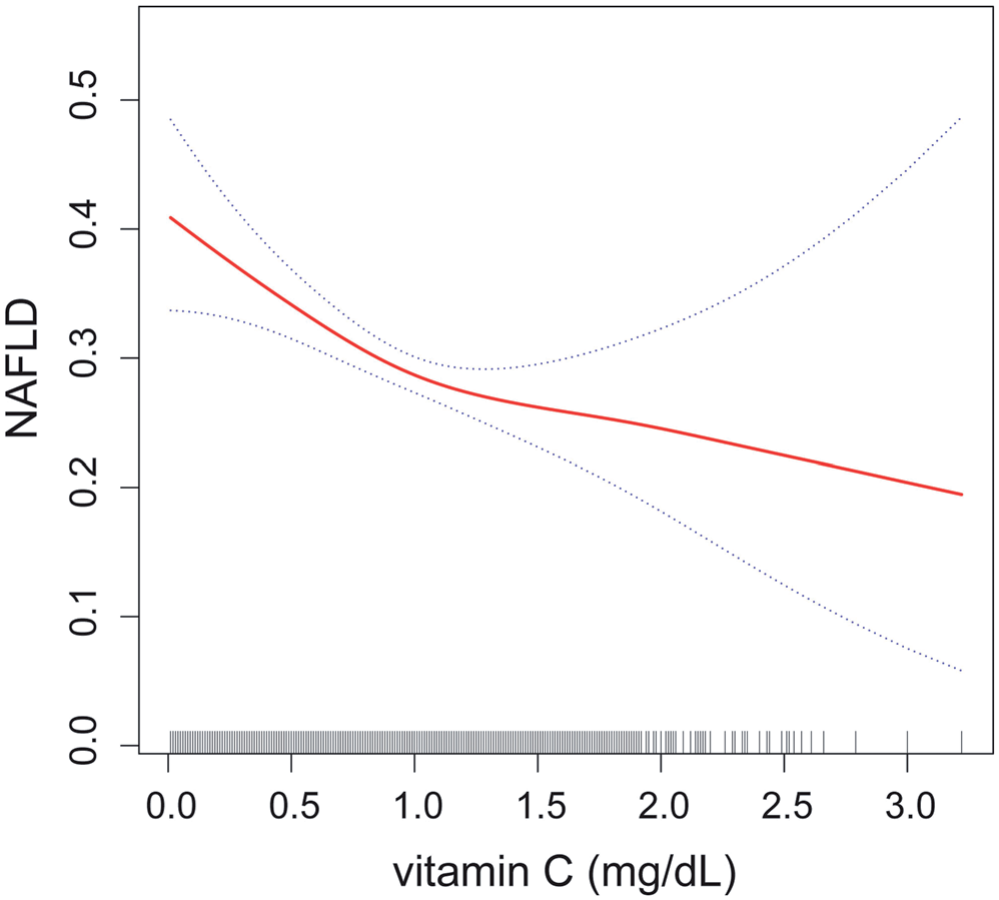
The relationship between serum vitamin C and the risk of NAFLD. A non-linear relationship between serum vitamin C and the risk of NAFLD was observed after adjusting for age, gender, race, education level, family PIR, body mass index, hypertension, diabetes, total cholesterol, triglyceride, smoke state, vitamin A, vitamin E. NAFLD, non-alcoholic fatty liver disease; PIR, poverty-to-income ratio.

The correlation between NAFLD and serum vitamin C was analyzed by the logistic regression analysis. The data with and without adjustment are presented in Table 2. An association could be seen clearly between continuous serum vitamin C and decreased risk of NAFLD in an unadjusted model (OR: 0.490, 95% CI: 0.413-0.581, *P* < .001), in a minimally adjusted model (with adjustment of age, gender, race, education level, and family PIR) (OR: 0.436, 95% CI: 0.362-0.524, *P* < .001) and in a fully adjusted model (with adjustment of age, gender, race, education level, family PIR, smoke status, BMI, hypertension, diabetes, total cholesterol, triglyceride, vitamin A, and vitamin E) (OR: 0.664, 95% CI: 0.512-0.860, *P* = .002). When the serum vitamin C level was divided into quartiles (Q1, Q2, Q3, and Q4), it was found that Q2, Q3, and Q4 were negatively correlated with the risk of NAFLD compared with the lowest quartile OR = 0.745, 95% CI: 0.566-0.982, *P* = .036; OR = 0.678, 95% CI: 0.508-0.905, *P* = .008; OR = 0.639, 95% CI: 0.467-0.875, *P* = .005, respectively. Trend test showed statistical significance (*P *< 0.001) in Table 2. As serum vitamin C was a continuous variable, a smoothing function was further used to explore the non-linear relationship. As depicted in Figure 2, the relationship was non-linear between vitamin C and risk of NAFLD after potential confounders were adjusted. Based on a 2-piecewise linear regression model, it was established that 0.92 was the inflection point, and a negative correlation was exhibited between serum vitamin C levels and the risk of NAFLD to its left (OR: 0.451, 95% CI: 0.288-0.706, *P* = .001). However, there was a lack of significant correlation between the risk of NAFLD and serum vitamin C levels to the right of the inflection point (OR: 0.996, 95% CI: 0.632-1.572, *P* = .988) (Table 3).

### Stratified Analysis by Potential Effect Modifiers

**Figure 3.**
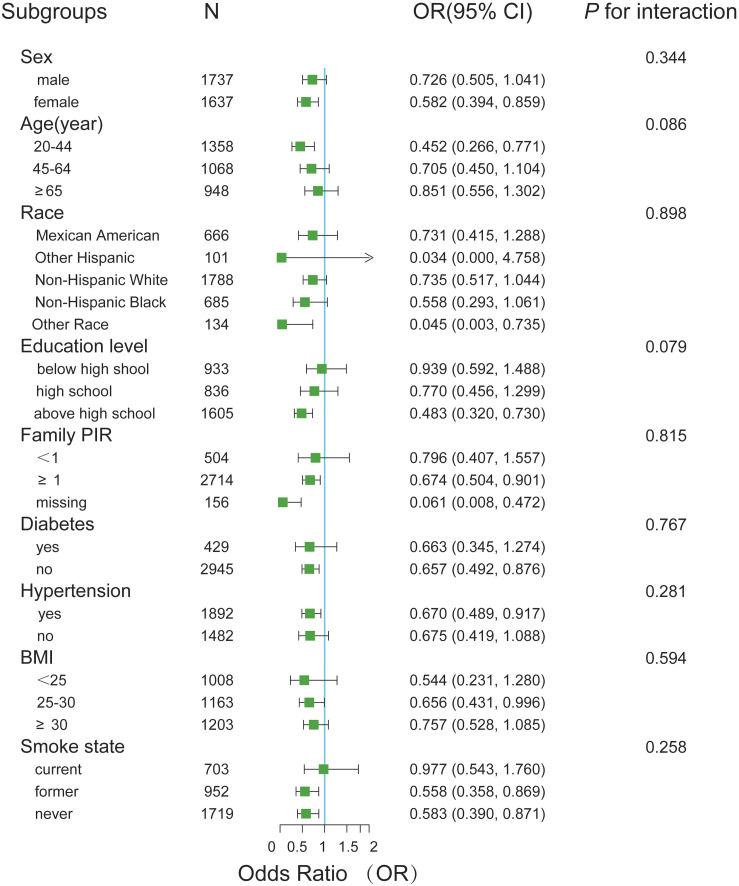
Forest plots of the association between serum vitamin C and the risk of NAFLD in various subgroups. Adjust for age, gender, race, education level, family PIR, body mass index, hypertension, diabetes, total cholesterol, triglyceride, smoke state, vitamin A, vitamin E, except for the stratifying variable. NAFLD, non-alcoholic fatty liver disease; PIR, poverty-to-income ratio.

Stratified analyses were further carried out using logistic regression to scrutinize the impact of vitamin C on NAFLD in different subgroups. The models adjusted all potential confounders except the confounders used for stratification itself. The results showed that none of the variables, including sex, age, race, family PIR, education level, smoking status, BMI, hypertension, diabetes, significantly modified the correlation between the risk of NAFLD and serum vitamin C levels, which implied the stability of this association among different groups (all *P *for interaction >.05) (Figure 3).

## Discussion

The aim of this study is to inspect the correlation between vitamin C and the risk of NAFLD in the US population, and a non-linear relationship was discovered. The turning point for serum vitamin C was 0.92 mg/dL. There was a negative correlation between serum vitamin C and risk of NAFLD when serum vitamin C level was below 0.92 mg/dL. However, the correlation became insignificant when vitamin C values exceeded this threshold. To our knowledge, this study is the first time that a correlation was discovered between serum vitamin C levels and risk of NAFLD with a threshold.

Oxidative stress, which contributes to inflammation, lipid peroxidation, and activation of hepatic stellate cells, has a major part to play in liver injury, the disease onset, and progression of NAFLD.^21^ Vitamin C is considered to be a powerful antioxidant owing to its ability to scavenge free radicals and reduce oxidative stress in vivo.^22^ Data from guinea pigs demonstrated that vitamin C deficiency resulted in accelerated dyslipidemia and an increase in oxidative stress markers in the liver, while an increased vitamin C intake lowered the quantity of lipid accumulation and alleviated the severity of dyslipidemia in hepatocytes.^23^ Another study also established that vitamin C treatment in rats with diet-induced non-alcoholic steatohepatitis led to reduced circulating liver enzymes, hepatocellular ballooning, and inflammation.^24,25^ Those experiment studies highlight the fact that vitamin C plays a protective role against NAFLD, and recovering the optimal serum vitamin C level could be a therapeutic approach to managing NAFLD.

Several epidemiologic studies have looked into the correlation between vitamin C and NAFLD, but the results remain inconclusive. A recent multivariate analysis on 3741 US adults showed a significant negative relationship between dietary intake of vitamin C and NAFLD among adults who were middle-aged or over, particularly among the non-obese male population.^13^ Another case–control study in Korea suggested that males with lower vitamin C intake than 66 mg/day were 4.23 times more likely to develop NAFLD.^12^ Vos et al^14^ reported a relationship between decreased serum vitamin C levels and increased hepatic ballooning in pediatric NAFLD patients. In line with the previous finding, the results of a 12-month pilot study conducted by Kawanaka et al^26^ found that the combination of vitamin E and vitamin C supplements achieved a significant improvement of liver fibrosis and serum alanine aminotransferase level in non-alcoholic steatohepatitis patients. However, another study involving a small sample of 74 NAFLD patients and 27 healthy controls concluded that dietary intake and serum levels of vitamin C were at similar levels between NAFLD patients and healthy controls.^16^ Similarly, Madan et al^27^ did not establish any differences in the serum vitamin C concentrations between 23 healthy controls and 29 NAFLD patients. Also, no correlation was found between serum vitamin C with either hepatic fibrosis or inflammation.^[Bibr b27-tjg-34-2-148]^ In the present study, however, it was observed that vitamin C serum concentrations were negatively correlated with the risk of developing NAFLD. This difference may be partially driven by small sample size in previous studies, which might have led to little power to disclose the associations. Furthermore, the difference among study designs and adjustment for different confounders of the study are other potential explanations.

Interestingly, it was noticed that the association was non-linear between the serum vitamin C level and risk of developing NAFLD; in addition, the negative correlation was only observed when serum level of vitamin C was lower than 0.92 mg/dL. Such a non-linear effect of vitamin C was also reported in a previous study,^28^ where the authors found an “L”-shaped association between serum vitamin C and peripheral arterial disease risk in active smokers.^28^ The mechanism behind the inflection point is not clear. However, this threshold effect has important clinical significance as it provides a valuable clue to find a serum vitamin C reference value, which can be used for guiding vitamin C supplements and reducing the risk of NAFLD in the US population.

Our study has several strengths. (1) The data of our study are based on a large nationally representative survey in the United States, which strengthened the statistical credibility and reliability. (2) Smooth curve fittings were used to explore potential non-linear relationships. This method is able to find the exact association when there is a possible threshold effect. (3) The stratification and interaction effect analyses in this study make better use of data and could generate a conclusion that remains valid in different subgroups. However, certain limitations of this study should be recognized. Firstly, NAFLD was diagnosed depending on USFLI, rather than by ultrasonography, which may underrate the true prevalence of NAFLD. Furthermore, as patients with steatogenic medication history or chronic liver disease such as autoimmune hepatitis and inherited metabolic liver disease were not excluded, the prevalence could be falsely calculated as higher. A further prospective study should be taken into account to avoid miscalculation of the population. Secondly, the present study could not supply any evidence of causality as a cross-sectional study; therefore, large prospective cohort studies are needed. Thirdly, the bias caused by other potentially unadjusted confounding factors was not excluded. In addition, our analyses were performed on an American population. Thus, further independent validation is still needed to examine whether the conclusion is applicable to populations in other regions. Last but not least, while liver fibrosis played an important role in the prognosis of NAFLD, our study failed to elucidate the relationship between vitamin C and the risk of liver fibrosis, which was worth discussing. Therefore, further studies are needed to shed light on this relationship.

To summarize, in the present study, we found a non-­linear association between serum vitamin C levels and the risk of developing NAFLD. The serum levels of vitamin C were negatively correlated with the risk of developing NAFLD when it is below 0.92 mg/dL. This work contributes to existing knowledge of NAFLD by providing novel insights into the potential therapeutic effect of vitamin C, thereby further advancing this field of research.

## Figures and Tables

**Table 1.** Basic Characteristics of the Study Population

Variable	Patients Without NAFLD (n = 2355)	Patients With NAFLD (n = 1019)	*P*
Sex (n, %)			<.001
Male	1138 (48.323)	599 (58.783)	
Female	1217 (51.677)	420 (41.217)	
Age [mean ± SD] (year)	49.372 ± 19.206	54.938 ± 17.233	<.001
Race (n, %)			<.001
Mexican American	359 (15.244)	307 (30.128)	
Other Hispanic	75 (3.185)	26 (2.552)	
Non-Hispanic White	1264 (53.673)	524 (51.423)	
Non-Hispanic Black	554 (23.524)	131 (12.856)	
Other race	103 (4.374)	31 (3.042)	
Education level (n, %)			<.001
Less than high school	583 (24.756)	350 (34.347)	
High school	569 (24.161)	267 (26.202)	
Above high school	1203 (51.083)	402 (39.450)	
Family PIR (n, %)			.430
PIR < 1	341 (14.480)	163 (15.996)	
PIR ≥ 1	1901 (80.722)	813 (79.784)	
Missing	113 (4.798)	43 (4.220)	
Smoking state (%)			
Current	500 (21.231)	203 (19.921)	<.001
Former	608 (25.817)	344 (33.759)	
Never	1247 (52.952)	472 (46.320)	
BMI [mean ± SD] (kg/m^2^)	26.740 ± 5.199	33.798 ± 6.734	<.001
Waist circumference [mean ± SD] (cm)	93.143 ± 12.427	112.564 ± 14.179	<.001
Hypertension (n, %)			<.001
Yes	1134 (48.153)	758 (74.386)	
No	1221 (51.847)	261 (25.614)	
Diabetes mellitus (%)			<.001
Yes	173 (7.346)	256 (25.123)	
No	2182 (92.654)	763 (74.877)	
Total cholesterol [mean ± SD] (mg/dL)	196.966 ± 42.397	201.897 ± 43.492	.002
Triglycerides [median (IQR)] (mg/dL)	102.00 (74.000-145.000)	163.000 (114.000-229.5000)	<.001
Vitamin C [median (IQR)] (mg/dL)	0.990(0.680-1.240)	0.830(0.490-1.125)	<.001
Vitamin A [mean ± SD] (µg/dL)	59.826 ± 16.771	62.166 ± 18.108	<.001
Vitamin E [median (IQR)] (µg/dL)	1150.000 (914.000-1492.000)	1220.000 (975.500-1607.000)	<.001
Gamma-glutamyl transferase [median (IQR)] (IU/L)	17.000 (13.000-24.000)	30.000 (22.000-45.000)	<.001
Fasting insulin [median (IQR)] (pmol/L)	38.220 (25.080-54.060)	105.900 (75.990-151.080)	<.001
Fasting glucose [mean ± SD] (mg/dL)	98.979 ± 23.273	123.653 ± 48.871	<.001

NAFLD, non-alcoholic fatty liver disease; BMI, body mass index; IQR, interquartile range; SD, standard deviation; PIR, poverty-to-income ratio.

**Table 2.** The Association Between Vitamin C (mg/dL) and NAFLD

	Non-adjusted OR (95% CI), *P* value	Adjusted I OR (95% CI), *P* value	Adjusted II OR(95% CI), *P* value
Serum vitamin C (mg/dL)	0.490 (0.413, 0.581), <.001	0.436 (0.362, 0.524), <.001	0.664 (0.512, 0.860), .002
1(≤0.60)	Reference	Reference	Reference
Q2 (0.61-0.94)	0.768 (0.628, 0.938), .009	0.735 (0.595, 0.907), .004	0.745 (0.566, 0.982), .036
Q3 (0.95-1.20)	0.587 (0.478, 0.720), <.001	0.526 (0.423, 0.652), <.001	0.678 (0.508, 0.905), .008
Q4 (≥1.21)	0.416 (0.335, 0.516), <.001	0.361 (0.287, 0.455), <.001	0.639 (0.467, 0.875), .005
*P* for trend	<.001	<.001	<.001

Non-adjusted model; Adjust model I: adjust for age, gender, race, education level, family PIR; Adjust model adjust II: adjust for age, gender, race, education level, family PIR, body mass index, hypertension, diabetes, total cholesterol, triglyceride, smoke state, vitamin A, vitamin E.

NAFLD, non-alcoholic fatty liver disease; OR, odds ratio; PIR, poverty-to-income ratio.

**Table 3.** Threshold Effect Analysis of Serum Vitamin C on NAFLD Using the 2 Piecewise Linear Regression Model

NAFLD	Adjusted OR (95% CI), *P* value
Fitting by the standard linear model	0.664 (0.512, 0.860), .002
Fitting by the 2-piecewise linear model	
Inflection point	0.92
Serum vitamin C < 0.92 (mg/dL)	0.451 (0.288, 0.706), .001
Serum vitamin C > 0.92 (mg/dL)	0.996 (0.632, 1.572), .988
Log likelihood ratio	0.039
